# Intravenous immunoglobulin for cutaneous chronic graft-versus-host disease

**DOI:** 10.1016/j.jdcr.2025.10.039

**Published:** 2025-10-28

**Authors:** Tien-Heng Chang, Trenton Greif, Jason Nayar, Adela R. Cardones

**Affiliations:** a Division of Dermatology, Department of Internal Medicine, University of Kansas Medical Center, Kansas City, Kansas; b University of Kansas School of Medicine, University of Kansas Medical Center, Kansas City, Kansas

**Keywords:** chronic graft-versus-host disease (cGVHD), hematopoietic stem cell transplantation (HSCT), immunosuppressive agents, intravenous immunoglobulin (IVIG)

## Introduction

Chronic graft-versus-host disease (cGVHD) is a morbid condition for which approved therapies are limited. Currently, no consensus has been reached regarding optimal treatment for cGVHD refractory to steroid or other treatments.^1^ Herein, we present a patient with refractory cutaneous cGVHD successfully treated with intravenous immunoglobulin (IVIG) therapy.

## Case

**Fig 1:**
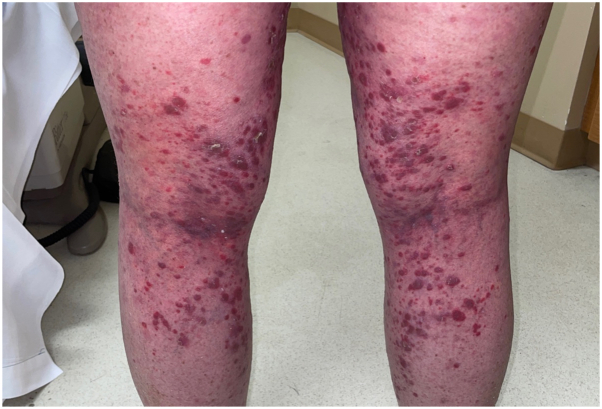
Erythematous and violaceous flat topped scaly round papules coalescing into plaques on the posterior lower extremities, consistent with cutaneous chronic graft-versus host disease (cGVHD).

**Fig 2:**
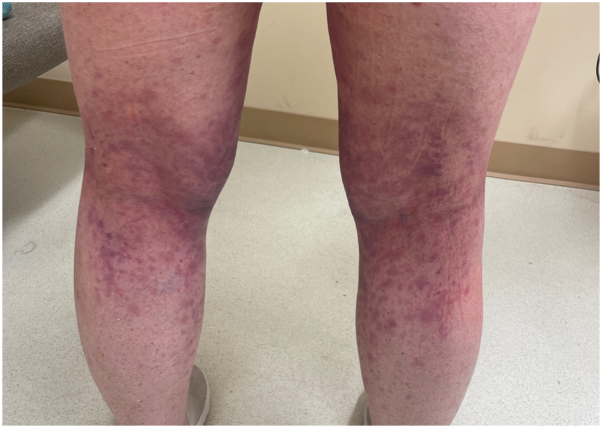
Improvement and resolution of papules and plaques 2 weeks after initiation of high dose IVIG therapy. *IVIG*, Intravenous immunoglobulin.

A female in her 60s presented with rapidly progressing and extensive cutaneous eruption that was consistent with cutaneous lichen planus-like cGVHD. She has a history of adult xanthogranulomas diagnosed 7 years prior. She was diagnosed with chronic myelomonocytic leukemia and underwent allogeneic hematopoietic stem cell transplantation from a haploidentical related donor 2 years prior. Her post-transplant course was complicated by acute GVHD of the lungs, liver, and gastrointestinal (GI) tract, followed by cutaneous cGVHD. She developed a rash on bilateral lower extremities 3 months after transplantation, consisting of flat-topped, erythematous to violaceous scaly papules coalescing into larger plaques (Fig 1). Biopsy of the left medial calf at the time demonstrated vacuolar interface reaction with epidermal dysmaturation, hemorrhage, and trace lymphocytic inflammation. The rash continued to worsen with involvement of the face, eyes, oral mucosa, trunk, bilateral upper and lower extremities. Total body surface area involvement was greater than 40%. She also developed recurrence of large, smooth, yellowish orange plaques on her arms, legs, chest, abdomen, and back, which was consistent with xanthogranulomatosis. Due to progression of her cGVHD in the skin, eyes, mouth, lungs, liver, and GI tract, she received oral prednisone taper beginning at 150 mg daily for 7 months, oral ruxolitinib 10 mg twice daily for 3 months, oral belumosudil 200 mg twice daily for 4 months, extracorporeal photopheresis every 2 weeks for 3 months, and 1 cycle of intravenous rituximab 700 mg. Due to persistence and progression of her skin disease, high dose (1 g/kg intravenous on 2 consecutive days) IVIG was added to her treatment regimen, which at that time included oral belumosudil 200 mg twice daily, oral prednisone 60 mg alternating with 20 mg every other day, and photopheresis every 2 weeks. She had rapid improvement of her cutaneous symptoms and xanthogranulomas at clinical follow-up 2 weeks later (Fig 2). The patient continued on monthly high-dose IVIG, belumosudil, and began taper of prednisone. Photopheresis was discontinued about 1 month after starting high-dose IVIG. Six months after initiation of high-dose IVIG, she had only 2% to 3% BSA involvement of cutaneous cGVHD. At 10 months, prednisone taper was completed and patient’s cutaneous cGVHD was successfully treated with IVIG infusions. One year later, she continues on monthly IVIG with belumosudil and has no active cGVHD involvement.

## Discussion

Corticosteroids remain first-line systemic therapy for cGVHD, but this can be associated with significant acute and long-term toxicities. Second-line treatment requires an empirical approach of available agents such as calcineurin inhibitors, extracorporeal photopheresis, or newer Food and Drug Administration approved therapies such as ruxolitinib, a Janus kinase inhibitor that acts by inhibiting the signal transduction and activation of transcription pathway; belumosudil, an inhibitor of Rho-associated coiled-coil-containing protein kinase 2 (ROCK2); imatinib, an inhibitor of several tyrosine kinases with inhibitory activity against platelet-derived growth factor; and ibrutinib, an irreversible inhibitor of both Bruton’s tyrosine kinase and IL-2-inducible kinase, but these have limitations as well.^1^^,^^2^ The complexity of the pathophysiology of cGVHD likely warrants a multi-pronged therapeutic attack, rather than monotherapy. The immunomodulatory properties of IVIG may be due to its ability to reduce pathologic autoantibodies. In autoimmune bullous dermatoses such as pemphigus vulgaris, it is hypothesized that IVIG saturates neonatal Fc receptors, resulting in accelerated degradation of pathogenic autoantibodies. In dermatomyositis, the Fcγ fraction of IVIG was shown to bind C3b, preventing the formation of the complement membrane attack complex.^3^^,^^4^ IVIG has also been noted to prevent the development of acute GVHD.^5^ In contrast to other immunomodulatory agents, IVIG has the added advantage of not being globally immunosuppressive and may be a useful adjunct when multiple immunosuppressive treatments are already on board. Adjunct therapy with high dose IVIG may be an ideal option for patients with refractory lichenoid or sclerodermatous cGVHD of the skin who are able to tolerate the infusions, and when avoidance of exacerbating immunosuppression is desired. In the meantime, further studies on the benefits of IVIG therapy as first of second-line adjunctive therapy for cutaneous and other organ involvement of cGVHD and are warranted.

## Conflicts of interest

None disclosed.
